# Learnings and challenges to deploy an interprofessional and independent medical education programme to a new audience

**DOI:** 10.1080/21614083.2017.1400857

**Published:** 2017-11-12

**Authors:** Mieke L. Van Driel, Treasure M. McGuire, Richard Stark, Patrice Lazure, Tina Garcia, Lisa Sullivan

**Affiliations:** ^a^ Primary Care Clinical Unit, Faculty of Medicine, University of Queensland, Brisbane, Australia; ^b^ School of Pharmacy, University of Queensland, Brisbane, Australia; ^c^ Faculty of Health Sciences & Medicine, Bond University, Gold Coast, Australia; ^d^ Mater Pharmacy Services, Mater Health Services, Brisbane, Australia; ^e^ Neurology Department, Alfred Hospital, Melbourne, Australia; ^f^ Department of Medicine, Monash University, Melbourne, Australia; ^g^ AXDEV Group, Brossard, Canada; ^h^ In Vivo Academy, Surry Hills, Australia

**Keywords:** interprofessional education, independent medical education, continuing medical education, continuing pharmacy education, continuing professional education, outcomes assessment, headaches

## Abstract

The importance of interprofessional education (IPE) in continuing medical education and professional development has long been recognised by health organisations and academic societies, benefiting not only patient outcomes and interprofessional relationships but also overall health systems and workforce shortage. We report on the outcomes of an Australian IPE activity on medication-overuse headache (MOH) with general practitioners (GPs) and community pharmacists as learners. The design of the activity, which followed the predisposing–enabling–reinforcing instructional framework by Green and Kreuter, aimed to: (1) improve knowledge and foster a willingness in GPs and pharmacists to work collaboratively to enhance the prevention, diagnosis and management of MOH; and (2) address their educational gap by demonstrating the utility of a blended learning IPE strategy on MOH. Integrated into the activity was an assessment of its effectiveness and impact to instil change in the participants’ knowledge of MOH, attitude and willingness to treat, and clinical practice behaviours of GPs and pharmacists to work together. The learners gained knowledge and confidence in diagnosing and managing MOH and in their ability to educate patients. The IPE approach suited the activity and was valued by the participating GPs and pharmacists, who seldom experience such learning formats. However, for educational providers in Australia, developing and deploying an independent medical education (IME) programme can be challenging. Providers of IMEs need to be aware of the potential pitfalls when competing with pharmaceutical-company-sponsored and delivered programmes.

## Introduction

### Interprofessional education and collaboration

The World Health Organization (WHO) recognises the importance of interprofessional collaboration in education and practice to mitigate the shortage of global health workforce, to strengthen health systems and improve patient outcomes [1]. The Society for Academic Continuing Medical Education describes interprofessional education (IPE) as “an intervention where the members of more than one health or social care (or both) profession learn interactively together, for the explicit purpose of improving interprofessional collaboration or the health/well-being (or both) of patients/clients” [2, p. S66]. A Cochrane review has shown IPE to have positive outcomes in the areas of diabetes care, emergency department culture, procedures and patient satisfaction, team behaviour in operating rooms, care management in cases of domestic violence and mental health practitioner competencies [3].

Traditionally, professional learning in the health sciences occurs in monodisciplinary educational systems, but global trends as identified by the WHO and the need for new models of care due to the growing prevalence of chronic conditions led to reforms in health professional education [4]. In Australia, the national standards body for medical education, training and continuing professional development (CPD), the Australian Medical Council, requires a range of teaching and learning approaches, one of which is “working with interdisciplinary and interprofessional teams” [5, p. 12]. And while many universities include IPE and interprofessional learning in their health and social care programmes, the content is general in nature; learning outcomes, including benefits to patients, are not formally assessed [4,6]. In countries such as the US and Canada where learning outcomes are routinely assessed, interprofessional CPDs have been shown to improve the participants’ understanding of the roles of different professions, resulting in improved respect and collaboration [7,8]. In 2016, Dunston et al. [9] submitted a report to the Australian government recommending embedding IPE into accreditation and CPD requirements for professional registration.

### MOH in primary care

Medication-overuse headache (MOH) and other recurrent headaches are a public health concern because of the disability and ill-health they cause in 1–2% of the population [10]. However, more worrying is that people affected are primarily self-treating without consulting a health care professional. Poor public awareness and lack of knowledge among healthcare providers have been identified as barriers to effective care [11]. Peters et al. [12] highlighted the importance of healthcare practitioners being well informed about diagnosis and management of headaches, especially GPs because of their long-term relationship with patients. In addition, Giaccone and colleagues [13] showed that based on worldwide data, community pharmacists can also play a crucial role in the management of chronic headaches, particularly due to their knowledge of prescription and over-the-counter (OTC) medications and their easy access to patients. However, effective outcomes are only possible with “adequate and continuous training on both the management of therapies and the relationship with the physicians and/or patients” [12, p. S4].

### Objectives

From a clinical perspective, we aimed not only to enhance knowledge, but also to foster a positive attitude in GPs and pharmacists towards working collaboratively to enhance the prevention, diagnosis and management of MOH. Our educational aim was therefore to address this educational gap by demonstrating the utility of a blended learning IPE strategy on MOH, delivered nationwide to GPs and pharmacists in the primary care setting.

## Methods

Using broad theories of constructivism and behaviourism to cultivate a collaborative and authentic online blended learning, interprofessional experience with intended behavioural change [14], we designed and deployed the programme, Medication Overuse headache: educaTion in dIagnosis, preVention, mAnagement and paTient Education (MOTIVATE). The programme arose from a grant received by In Vivo Academy (IVA) – an Australian-based, not-for-profit, accredited education provider to the Royal Australian College of General Practitioners (RACGP).^1^ Recruiting participants all over Australia, MOTIVATE extended the IPE model to a primary care audience of GPs and community pharmacists. It tackled an often under-recognised and poorly managed medication-induced condition with the key stakeholders who could effect change.

In this paper, we report on the interprofessional nature as well as the instructional framework and blended design of MOTIVATE and assessed the areas in the programme that worked well for the education of these healthcare professionals involved in the care of people with headaches, and the improvements that may guide future IPE programmes. Learnings from this activity are expected to benefit other CME providers, faculty, learners and funders of independent medical education (IME) programmes.

### Needs assessment

Between September 2013 and February 2014, IVA conducted two needs assessment surveys: one for GPs and another for pharmacists. The GP survey was distributed to about 3000 GPs in IVA’s database, while the pharmacist survey was advertised to approximately 5000 members of the Australian College of Pharmacy (ACP) through press releases in their newsletters distributed in December 2014, January and February 2015.

In the GP survey, we explored the prevalence of different types of headaches in the respondents’ own practices and their confidence in diagnosing and managing patients with headache. We also assessed their perceived knowledge of the prevalence and incidence of MOH, their knowledge of the International Headache Society guidelines on the prevention, diagnosis and management of MOH and their level of confidence in their ability to identify MOH or the risk of MOH.

The pharmacist survey explored the pharmacists’ knowledge of MOH, their ability to detect warning signs of MOH and their confidence to provide information and advice to their customers about MOH and motivate them to consult their GPs.

While the results of the needs assessment surveys were used to guide MOTIVATE’s programme design and structure, a detailed report is beyond the scope of this paper.

### Programme design and structure

Based on the above needs assessments, GPs and pharmacists would benefit from an IPE CPD activity on MOH. IVA and an expert steering committee, composed of primary and specialist care providers, planned and developed the IPE CPD programme MOTIVATE using the criteria of the RACGP for an active learning module (ALM). This “provide[s] structured, quality education opportunities directed to achieving demonstrable changes in the performance, knowledge, skills, behaviours, and attitudes” [15, p. 32] The ALM design is similar to the predisposing–enabling–reinforcing instructional framework by Green and Kreuter [16,17] that facilitates the adult learning process through a learning cycle of: self-reflection, planning, action, review and again, planning. As such, it consists of: (a) a predisposing activity that provides an opportunity for participants to reflect on their current clinical practice; (b) a structured learning activity of at least 6 hours, covering both a *person approach* to enhance professional competence (behaviour, attitude, skills and knowledge) and a *system approach* that focuses on team and procedural processes to safeguard patient safety; and (c) a reinforcing activity that consolidates learning [14]. This also integrates well with the five-stage physician learning model described by Moore et al. [16]: (1) recognising an opportunity for learning; (2) searching for resources for learning; (3) engaging in learning to address an opportunity for improvement; (4) trying out what was learned; (5) incorporating what was learned. The integrated outcomes assessment was structured based on Moore’s framework, which described seven levels of outcomes: 1 – Participation; 2 – Satisfaction; 3 – Knowledge; 4 – Competence; 5 – Performance; 6 – Patient health; 7 – Community health [16].

The MOTIVATE programme’s structure and content were as follows:

- Predisposing activity/pre-work – a short questionnaire (available online or paper-based), which required participants to recall a patient/client who presented with frequent headaches or often sought pain medication for headaches in their practice or pharmacy, and describe how headache histories were obtained, how these patients were diagnosed, treated, managed and whether they were referred for speciality care. This aimed to help the participants “recognise a teachable moment” [16, p. 5], and an interest to pursue the subsequent modules.
- Module 1: Fundamental concepts of MOH – a 2-hour online course, with case-stemmed, multiple-choice and short-answer questions, short videos of an MOH expert and a 20-item quiz to test their knowledge at the end. This module covered the epidemiology of MOH, the diagnostic criteria, patterns of MOH, managing withdrawals, identifying patients at risk and educating patients.
- Module 2: MOH: a case-study approach – a 2-hour evening meeting held in major cities in Australia; two of the 20 meetings were also made available as live or archived webinars. Facilitators at all Module 2 meetings and webinars were neurologists with interest and expertise in chronic headaches. This module allowed engagement with other participants and opportunities to try out what was learned from Module 1 in a safe environment of case-based learning. It also served as an opportunity to obtain feedback from the neurologist who stood as facilitator, thus learning the difference between “what they are doing and what they should do to improve” [16, p. 5] physician competence (Outcomes level 4 of the Moore framework) [10]. All participants received booklets, which included all the key points of the activity, and enough space to record feedback from facilitator and co-participants.
- Module 3: Back at practice – a 2-hour combination of online and practical application of learning how to obtain a comprehensive headache history. This module allowed participants to apply their learning in their practice. We provided them with key pieces of information for use at point of care such as MOH identification flowchart, quick references for motivational interviewing, MOH patient/client education checklist and headache history forms.
- Reinforcing activity/post-work – a combination of a practical activity and an online form, requiring participants to ask patients who presented with frequent headaches, migraine or possibly MOH, to complete a self-administered questionnaire, then have participants answer questions online. This activity required participants to reflect on the patients’ responses about their frequent headaches and their treatment, readiness to accept their condition and to shift treatment, and their fears and concerns about their condition, side effects, withdrawal and tolerance. This also allowed participants to reflect on the educational gaps that still need to be addressed, both for the healthcare professionals and the patients, and set off a continuous learning process and self-improvement.

The content of the programme was based on a review of the literature conducted by IVA staff and informed by experts in the field. A steering committee, consisting of a neurologist/headache specialist, a GP, two pharmacists and a professor of psychology, was formed to discuss the content and critically review all materials. Programme development and module creation took approximately 8 months. IVA finalised the contents for the online components and face-to-face meeting, wrote the promotional materials and invitations, and directly deployed the invitations to the targeted participant audience via fax and email.

MOTIVATE was approved by the RACGP Quality Improvement & Continuing Professional Development programme as an ALM and by the Australian College of Rural and Remote Medicine (ACRRM) as a Planned Reflective Professional Development activity. The three MOTIVATE modules were also accredited individually. The ACP also accredited the individual components of the programme.

The learning objectives of the MOTIVATE programme were to:

- define MOH including the agents that are likely to cause them based on the International Classification of Headache Disorders, 3rd edition (ICHD-III) diagnostic criteria [18];
- identify patients susceptible to MOH;
- develop integrated prevention and management skills, such that when MOH is recognised:
  - *pharmacists* are able to motivate patients to seek the help of GPs, and
  - *GPs* are able to develop an appropriate management plan and effectively communicate this to the patient; and
- put a system in place so that patients are made aware of their susceptibility or risk of MOH.

### Participants

GPs who have attended CPD activities previously developed by IVA, who agreed to be contacted for future activities and who practise in close proximity to the meeting venues were invited by email or fax between February and July 2015 to register for the MOTIVATE programme. Identified using a simple Google search, GP surgeries and pharmacies within a 10 km radius of a city meeting venue or up to within 100 km radius of a rural meeting venue (due to rural Australia’s vastness and sparse population) were sent fax invitations, and GPs and pharmacists interested in the programme were asked to return the faxed form or to register on the website, www.motivate.org.au. MOTIVATE was listed on the RACGP (https://www.racgp.org.au) and ACRRM (http://www.acrrm.org.au) websites, and advertised on the electronic newsletters distributed by the ACP to its members.

Registered participants were advised to complete the predisposing activity and online Module 1 before attending Module 2 or the 2-hour evening meeting in their area. After Module 2, they were instructed to log back into the programme website to complete Module 3 and the reinforcing activity. All participants were given a deadline of 30 September 2015 to complete the entire programme.

### Outcomes evaluation design, collection and analyses

Integrated into the MOTIVATE programme was an assessment of the effectiveness and impact of the programme to instil change in participant MOH knowledge, attitude and willingness to treat, and clinical practice behaviours. We evaluated the programme with a mixed-methods approach using responses from the predisposing activity (pre-work), evaluation forms, reinforcing activity (post-work) and a qualitative assessment, in which participants were independently contacted for interviews to obtain an in-depth understanding of changes implemented in clinical practice, and of facilitators and/or barriers to these changes. Participants provided informed consent (either written or online) for their survey or interview data to be used for evaluation and research purposes. The programme evaluation was approved by an independent Institutional Review Board (IRB) consistent with ethical principles outlined in the Australian National Statement on Ethical Conduct in Research Involving Humans (2007), to ensure anonymity of participants and confidentiality of the information collected (Protocol number 17: 206; IRB Services Ltd., a Chesapeake IRB company).

Participant data for outcomes assessment were collected at several points in the programme:

1. pre-assessment: baseline data from the predisposing activity forms;
2. immediate-post survey: participants completed an evaluation form upon completion of the full ALM programme to measure satisfaction, perceived achievement of the learning objectives and fulfilment of their learning needs, their intention to apply learnings to clinical practice, and self-reported confidence and change in knowledge compared with the needs assessment; and
3. 2-months-post qualitative assessment: eight participants who provided consent to be contacted after completing the programme were interviewed telephonically using a 45-minute semi-structured discussion guide to assess knowledge maintenance, self-reported confidence and self-reported application of learnings to clinical practice.

In addition, 30-minute semi-structured telephone interviews were held with members of the programme’s organising team, steering committee and a meeting facilitator as formative assessment to identify what worked well and what needed improvement in each of the components of the programme, particularly in the planning, development, deployment, recruitment and enrolment. Internal and external perceptions of the initiative and the collaborative process as a whole were also evaluated.

Control data were obtained via the pre-post evaluation design. Quantitative data were analysed using SPSS 22.0 software (SPSS, Chicago, IL) and reported as frequencies and cross-tabulations. Using a variation of thematic analysis (N-Vivo 7.0 software; QSR International, Cambridge, MA), qualitative data were analysed using a four-step approach: (1) codes identified based on literature review and the interviewer’s debriefing; (2) transcripts coded according to the developed coding structure; (3) new codes developed for data that did not fit the pre-defined codes; and (4) key emerging themes identified from the data [19,20]. Aggregate data collected through both quantitative and qualitative methods were triangulated, thus ensuring a more comprehensive report on the outcomes of the programme. Findings were then classified based on Moore’s outcomes framework described above [16].

## Results

### Participation (level 1)

**Figure 1.**
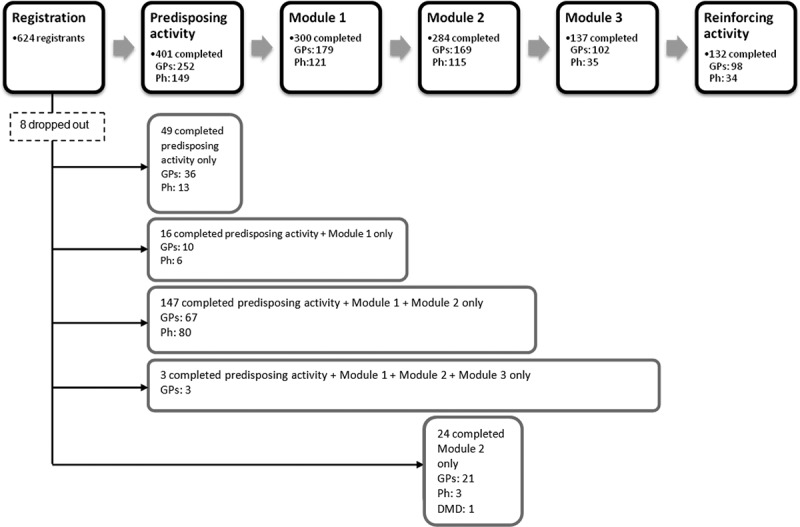
Registration, actual participation and completion of programme. (GP = general practitioners, Ph = pharmacists).

Between November 2014 and July 2015, IVA received a total of 624 registrations for the MOTIVATE programme across Australia (Figure 1). Three hundred participants completed both the predisposing activity and Module 1 (179 GPs, 121 pharmacists); of these, 147 proceeded to complete Module 2 (67 GPs, 80 pharmacists), 137 continued to Module 3 (102 GPs and 35 pharmacists), and 132 eventually completed the entire programme including the reinforcing activity (98 GPs and 34 pharmacists). Although we encouraged the sequential completion of the modules, we allowed 52 participants who have completed only the predisposing activity to attend the meeting (Module 2) and asked them to complete Module 1 after the meeting if they were interested in obtaining 40 Category 1 points; 25 participants attended only Module 2.

### Satisfaction and knowledge – self-reported survey (level 2 and levels 3a/3b)

**Figure 2.**
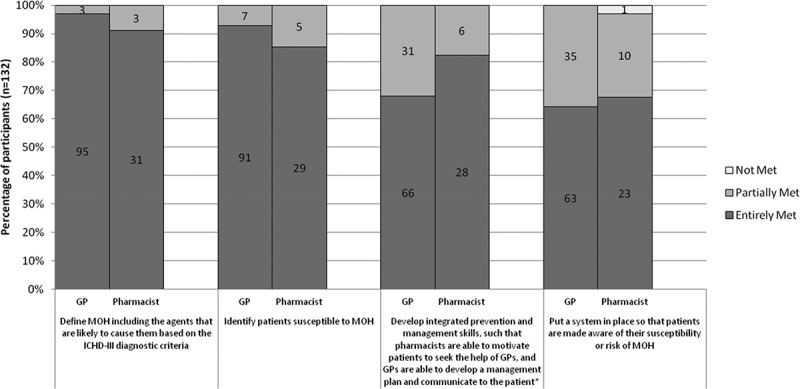
Perceived achievement of learning objectives. *One GP did not answer this part of the evaluation. Thus, the total is only 131 participants for this learning objective.

**Figure 3.**
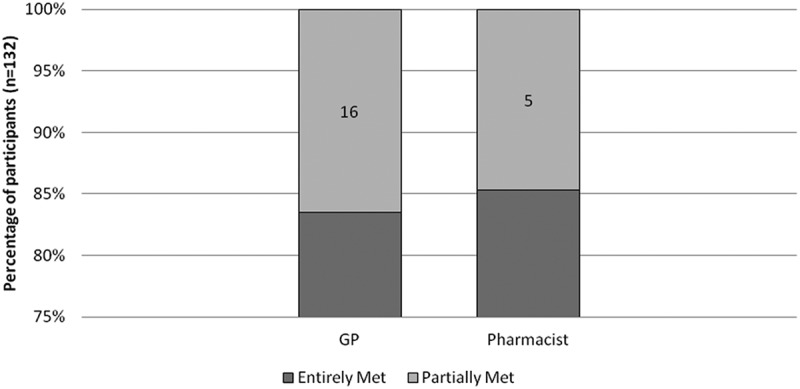
Degree to which learning needs of participants were met.

**Figure 4.**
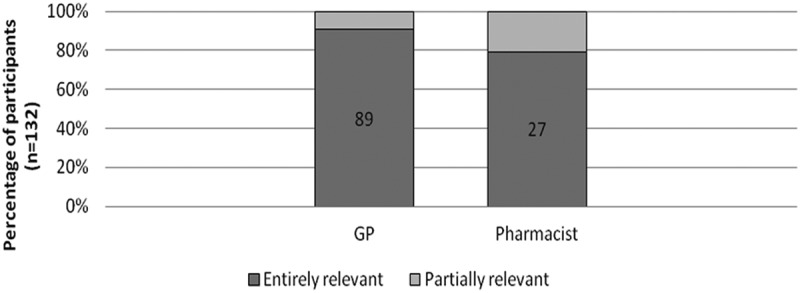
Degree of activity’s relevance to participants’ practice.

Based on the responses to the immediate-post survey, 80% of GPs (*n* = 98) and 82% of pharmacists (*n* = 34) on average felt that each of the learning objectives was fully met (Figure 2); 83% GPs and 85% of pharmacists reported that their learning needs were entirely met (Figure 3); and 91% of GPs and 85% of pharmacists rated MOTIVATE as entirely relevant to their practice (Figure 4).

Some of the verbatim feedback from the immediate-post survey with regard to satisfaction and knowledge include:

“I would like to thank everyone involved in preparing this comprehensive course which has been very educational it has changed my practice for the management of headache patients” – GP

“Thank you for this session, very informative and educational. I had been researching this topic myself and was excited when I received the fax/notification for this talk” – GP

“This was a great programme that made me think and was the best programme I have done for some time” – GP

“Excellent programme for [a] common topic” – GP

“I enjoyed the combination of pharmacist and GP participants” – GP

“Good for pharmacist learning” – pharmacist

“Publicity to involve GPs in the project was limited, which was a pity” – pharmacist

**Figure 5.**
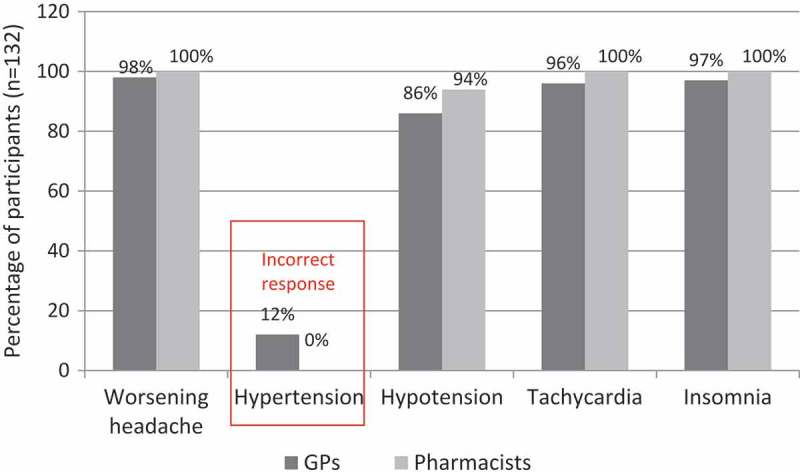
Assessment of change in knowledge (MOH withdrawal symptoms).

**Figure 6.**
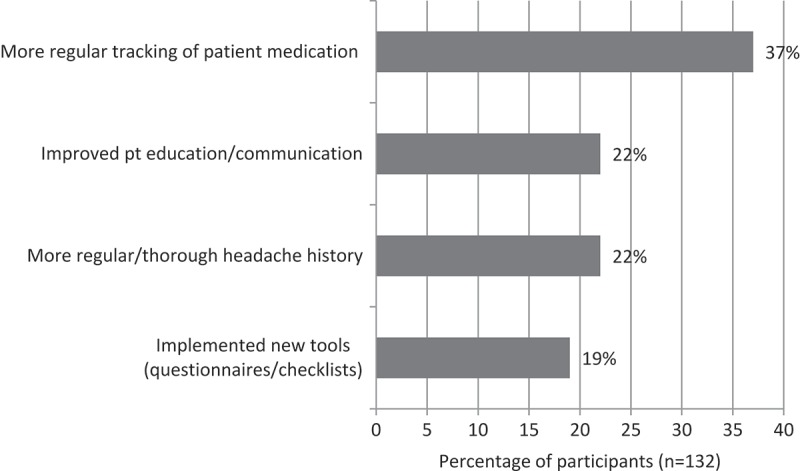
Changes most frequently reported by programme participants.

Assessment of changes in knowledge showed that 80% of all participants post-MOTIVATE participation correctly identified all four withdrawal symptoms of worsening headaches, hypotension, tachycardia and insomnia, compared with our needs assessment, which showed 50% of GPs reporting not so or not at all confident in knowledge of withdrawal symptoms. Improvement in knowledge was also indicated by a shift from 12% who scored incorrectly in the needs assessment to 0%, that is, no one gave an incorrect response to the symptoms of MOH (Figure 5). Around 98% of participants reported making at least one change to their practice, and four of the most frequently reported changes include: (1) more regular tracking of patient medication; (2) improved patient education and communication; (3) more regular/thorough headache history; (4) implemented new tools such as questionnaires and checklists (Figure 6).

**Figure 7.**
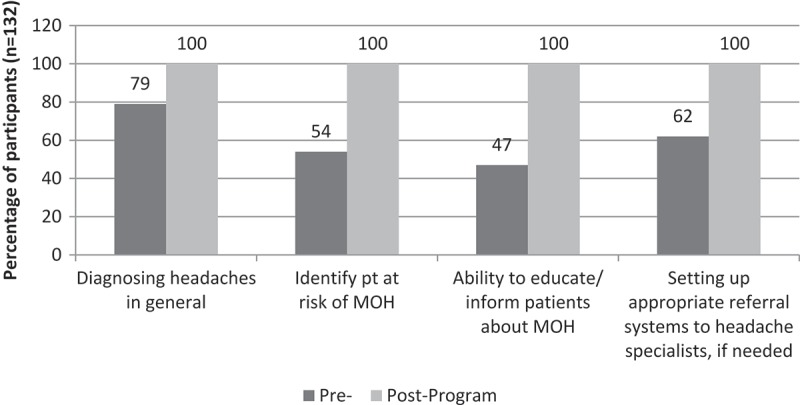
Levels of confidence after completing the entire programme.

Participants also reported increased confidence in diagnosing headaches in general, in identifying patients at risk of MOH, in their ability to educate or inform patients about MOH, and in setting up appropriate referral systems to headache specialists, if needed. (Figure 7)

### Competence (level 4)

Eight participants (7 GPs; 1 pharmacist) were interviewed 2 months after their completion of the programme to explore maintenance of knowledge, self-reported confidence and application of learnings to clinical practice. All participants who engaged in the 2-month post assessment reported increased awareness and knowledge of MOH after the programme.

“Before I entered the programme I really hadn’t thought very much about MOH, it was never mentioned in medical school” – GP

“I really didn’t dwell on it beforehand at all. I don’t know that I really warned people prior to doing the programme about the potential for the problem. Whereas now I do.” – GP

“I’d heard of the concept of MOH but I didn’t really know the details. What to describe to patients, what sort of things I should be looking for, what are the risk factors. So pretty much all of it was new to me. Changed the way that I look at analgesia.” – pharmacist

Self-reported confidence improved:

“I am more confident. Right now I am really more confident about my diagnosis.” – GP

Participants reported taking more thorough headache histories 2 months after attending the programme. A GP commented:

“I now take a basic headache history. I just quantify it more than I did before. So instead of vaguely ‘what medication do you take?’ I actually ask them how much and when and so on. That’s the main difference. And obviously warn them if they are taking OTC medications that it is not without risk and give them strategies so that they don’t fall into the trap.”

A pharmacist stated:

“Before I would not have picked [MOH] up whereas now I’m on the hunt for it. So when they say they’re using a lot of PRNs or a lot of analgesia I’ll say ‘How much are you using? If you stop taking it do you get rebound headaches?’ It’s given me something more to talk about with the patients.”

Participants commented that completing Module 2 (“A case-study approach”) and receiving feedback from the neurologist facilitator sufficiently addressed their learning needs. Especially, if MOH was not seen as a priority, completing Module 3 (“Back at practice”) and the reinforcing activity was considered unnecessary work. Some GPs also mentioned they did not need to complete the entire programme, as they already had enough points to meet their CPD requirements for the triennium.

### Formative assessment by the steering committee and organisers

The formative assessment revealed that a low completion rate by participants may be due to lack of awareness and prioritisation of MOH in primary care, some issues related to the current CME context in Australia, the lack of understanding by the target audience of the programme focus and participation requirements, technical difficulties with the online modules and perceived relevance to select programme modules to practice. (Table 1) In addition, the formative data also provided insights with respect to the challenges inherent in deploying an IME activity in Australia, notably, independent of any support by industry medical liaisons or sales representatives.

**Table 1.** Formative assessment of casualties.

Under-recognition of MOH in primary care	• Lack of awareness of MOH • Not seen as a priority issue
Issues related to current continuing medical education context in Australia	• Recruitment of participants for educational programmes traditionally done by industry representatives through their relationships with physicians • Challenging to get people to understand nature of the programme (i.e. Independent Medical Education) • Without this pre-defined network of potential participants, IVA required additional resources to identify/develop lists of participants
Low completion rate compared with the number of participants who registered and attended the face-to-face meeting (Module2)	• Due to the participants’ view that completing Module 2 and receiving feedback from the neurologist facilitator were enough and had already met their learning needs. • Some GPs do not feel the need to complete the entire programme as they have earned enough points to meet the RACGP requirements
Lack of understanding by target audience of the programme focus and participation requirements	• It was reported that invitation materials could have been clearer: ∘ The programme focus was not headaches but more specifically MOH ∘ Amount of effort required from participants (i.e. 6 h of learning plus predisposing and reinforcing activities) to earn 40 Category 1 points could have been better defined ∘ Description of interprofessional aspect of the programme could have been more clear • Completion of the programme requires participants to return to practice and apply programme learnings, but not all participants had the opportunity to apply programme learnings to an MOH patient in practice
Technical difficulties with the online modules	• Some course responses appeared to not register and participants would need to restart • Could have impacted participant’s willingness to continue to complete the programme
Perceived relevance of select programme modules to practice	• Steering Committee members reported a review of the final programme materials would have been beneficial to smooth out any remaining issues: ∘ A last review of the content would have allowed for the additional comments: ∘ Inclusion of more interactive components for the live session (Module 2) ∘ Adapting content of Module 1 to ensure its relevance for primary care providers

^1^The RACGP is a professional organisation of about 30,000 GPs in Australia. It supports GPs, GP registrars and medical students through education, training and research. (http://www.racgp.org.au/yourracgp/organisation/).

## Discussion

The MOTIVATE programme was designed to enhance insights and knowledge, and bring about change in clinical practice behaviours among GPs and pharmacists to work together in order to enhance prevention, diagnosis and management of MOH. Moreover, the programme was designed and deployed independently according to the parameters of an IME, and followed an IPE CPD model wherein GPs and pharmacists were trained together using the predisposing–enabling–reinforcing instructional framework.

The participants were found to have increased knowledge and improved confidence in diagnosing headaches in general, identifying patients at risk of MOH, including its likely causes based on the ICHD-III criteria and in identifying patients susceptible to MOH. They also gained confidence in their ability to educate or inform patients about MOH and setting up appropriate referral systems, if needed.

The response to MOTIVATE was positive, in terms of the relevance and uniqueness of the interprofessional nature of the activity. The outcomes analysis indicated that the activity’s objectives were fully met, that the activity was entirely relevant to the participants’ practice, and that participants’ learning needs were met. The participants recognised the value of the educational programme on a common condition such as chronic headache and the consequences of medication overuse if not detected. Both GPs and pharmacists welcomed the interprofessional nature of the activity.

The IPE approach suited the topic and the activity well, as it highlighted the roles and responsibilities of and between GPs and pharmacists. According to Pullon’s [21] research findings, working together for the common goal of patient health and recognising their respective skill sets leads to respect and interprofessional trust. Change in clinical practice behaviours as a result of IPE occurs when: (1) the education emphasises the participants’ common goals or experience; (2) encourages respect and trust; and (3) provides an opportunity to share differing opinions and experiences in a safe environment. This allows a coherent, collective decision rather than just a consensus to be reached [22]. These conditions were observed during the face-to-face meetings and self-reported by the participants, but we were unable to link this to actual change in clinical behaviours, for example by way of observations in patient care setting or charts.

While countries such as the Netherlands have a long tradition of GP–pharmacist IPE activities [23,24], these are not common in Australia, based on advertised educational activities both in the RACGP and ACP websites and also in comments we received from our participants. Because of the plethora of CPD activities offered to GPs, usually sponsored by pharmaceutical companies, GPs are accustomed to attending symposia and workshops where they earn CPD points to meet their respective college requirements. Australian pharmacists, on the other hand, do not have the same opportunities with most of their CPD activities being self-directed learning. The interaction and the collaboration among GPs, pharmacists and neurologists were therefore appreciated by all participants. Collaborations between GPs and pharmacists were also viewed favourably in studies outside Australia [19,20].

There were challenges with recruitment of participants, especially GPs. The pharmaceutical industry uses representatives who have personal relationships with doctors and pharmacists and use mailing lists to alert their clients to upcoming education opportunities. Without this type of assistance, we had to generate a list of the target group and send out invitations and connect with them effectively through simple, direct but attractive messaging. In the absence of face-to-face social marketing, this was challenging through emails and faxes.

We received a total of 624 registrations, but only 20% completed the entire programme; a large part of the attrition occurred after attending the face-to-face meeting. This may be due to some of the participants’ view that completing the programme after Module 2 (“A case-study approach”) and receiving feedback from the neurologist facilitator were sufficient to meet their learning and CPD requirements. If participants did not see MOH as a priority issue, completing Module 3 (“Back at practice”) and the associated reinforcing activity would be considered unnecessary work. Therefore, it is important to understand the needs of the target audience and develop a flexible programme that caters for different needs and interests.

To our knowledge, our study is the first to report on an IPE programme with GPs and pharmacists in Australia. However, there are some limitations to its generalisability, as the response rate decreased at each point of measurement due to attrition of the enrolled participants. But this attrition reflects a real-life scenario and provides pragmatic evidence. The qualitative data reveal interesting insights to understand the complex reality of pushing education out to health professionals in a context where a plethora of (often industry-sponsored) education is freely available.

Face-to-face meetings remain the most popular form of CPD activity in Australia despite the growing popularity of online learning courses. Yee et al. [25] reported that 83% prefer face-to-face lecture-based formats compared with 55% who prefer online self-education. However, in a large but sparsely populated country like Australia, a blended approach to learning is more financially viable and can be as effective as the traditional face-to-face modes. Blended learning has also consistently shown effectiveness in knowledge acquisition compared with no intervention at all [26,27]. It has demonstrated improvements in learning outcomes in graduate studies where curricula are based on adult learning theory [8,9,28,29]. However, no evidence, as yet, has demonstrated the extent to which deep learning can result from a short-term learning scenario, such as CPD activities where adult learning principles apply. Although CPD activities are required by all healthcare professional organisations in Australia, the learners are still the ones to decide and manage their own learning. For example, some participants perceived the online component of the programme to be difficult and so decided not to complete the online modules but only attended the face-to-face meeting.

Developing and deploying an IME programme in Australia, being a new concept, had its challenges. Even though the authors of this paper have many years of combined experience in education, training and CPD development, the MOTIVATE programme was a new learning experience. Independent education providers need to be aware of the potential pitfalls when competing with pharmaceutical-company-sponsored and delivered programmes. However, it is likely that the future of accredited CPD education in Australia will follow the example of other countries, where the concept of education planning and content will be conducted entirely independently of pharmaceutical industry involvement. The value of IPE is recognised not just by large organisations such as the WHO and governments, but by the learners themselves. Therefore, postgraduate continuing professional education should consider including all relevant professional disciplines in education activities.
